# Frequent Dietary Multi-Mycotoxin Exposure in UK Children and Its Association with Dietary Intake

**DOI:** 10.3390/toxins16060251

**Published:** 2024-05-28

**Authors:** Praosiri Charusalaipong, Margaret-Jane Gordon, Louise Cantlay, Nicosha De Souza, Graham W. Horgan, Ruth Bates, Silvia W. Gratz

**Affiliations:** 1 Rowett Institute, University of Aberdeen, Aberdeen AB25 2ZD, UK; p.charusalaipong.20@abdn.ac.uk (P.C.); m.j.gordon@abdn.ac.uk (M.-J.G.); l.cantlay@abdn.ac.uk (L.C.); r.slater@abdn.ac.uk (R.B.); 2 Biomathematics and Statistics Scotland (BioSS), Aberdeen AB25 2ZD, UK; nicosha.desouza@bioss.ac.uk (N.D.S.); g.horgan@abdn.ac.uk (G.W.H.)

**Keywords:** mycotoxin, biomonitoring, exposure, diet, children, risk assessment

## Abstract

Mycotoxins are potent fungal toxins that frequently contaminate agricultural crops and foods. Mycotoxin exposure is frequently reported in humans, and children are known to be particularly at risk of exceeding safe levels of exposure. Urinary biomonitoring is used to assess overall dietary exposure to multiple mycotoxins. This study aims to quantify multi-mycotoxin exposure in UK children and to identify major food groups contributing to exposure. Four repeat urine samples were collected from 29 children (13 boys and 16 girls, aged 2.4–6.8 years), and food diaries were recorded to assess their exposure to eleven mycotoxins. Urine samples (*n* = 114) were hydrolysed with β-glucuronidase, enriched through immunoaffinity columns and analysed by LC-MS/MS for deoxynivalenol (DON), nivalenol (NIV), T-2/HT-2 toxins, zearalenone (ZEN), ochratoxin A (OTA) and aflatoxins. Food diaries were analysed using WinDiet software, and the daily intake of high-risk foods for mycotoxin contamination summarised. The most prevalent mycotoxins found in urine samples were DON (95.6% of all samples), OTA (88.6%), HT-2 toxin (53.5%), ZEN (48.2%) and NIV (26.3%). Intake of total cereal-based foods was strongly positively associated with urinary levels of DON and T-2/HT-2 and oat intake with urinary T-2/HT-2. Average daily mycotoxin excretion ranged from 12.10 µg/d (DON) to 0.03 µg/d (OTA), and co-exposure to three or more mycotoxins was found in 66% of samples. Comparing mycotoxin intake estimates to tolerable daily intakes (TDI) demonstrates frequent TDI exceedances (DON 34.2% of all samples, T-2/HT-2 14.9%, NIV 4.4% and ZEN 5.2%). OTA was frequently detected at low levels. When mean daily OTA intake was compared to the reference value for non-neoplastic lesions, the resulting Margin of Exposure (MoE) of 65 was narrow, indicating a health concern. In conclusion, this study demonstrates frequent exposure of UK children to multiple mycotoxins at levels high enough to pose a health concern if exposure is continuous.

## 1. Introduction

Mycotoxins are highly toxic fungal metabolites that are frequently found in some food commodities, and mycotoxin contamination poses a major global food safety concern [1]. Mycotoxins are produced by a wide range of fungi on numerous food and feed crops, and global patterns of fungal infection are impacted by climate change and severe weather events [2,3]. As a result, human mycotoxin exposure is increased during years of severe fungal infection [4]. In temperate climate regions, *Fusarium* fungi pose a major problem and emerging risk for small-grain cereals, resulting in contamination with numerous mycotoxins, including trichothecenes and zearalenone (ZEN). Type B trichothecenes deoxynivalenol (DON) and nivalenol (NIV), as well as type A trichothecenes T-2 and HT-2 toxins (T-2/HT-2), are commonly found in raw cereals as well as cereal-derived foods such as bread, pasta and breakfast cereals [5,6,7]. In addition to free *Fusarium* mycotoxins, plant-derived mycotoxin glucosides and other modified forms frequently co-occur in the same commodities and can contribute to overall exposure [8]. *Aspergillus* fungi produce ochratoxin A (OTA) and aflatoxin B_1_ (AFB_1_), both of which have carcinogen properties. OTA is frequently found in cereal foods, coffee and grape products, while AFB_1_ contaminates ground nuts and maize [9,10]. To minimise exposure to mycotoxins in humans, the European Food Safety Authority (EFSA) has introduced maximum permitted levels for DON, ZEN, OTA and AFB_1_ in food commodities [11], and limits for T-2/HT-2 are currently being finalised. However, these levels aim to prevent highly contaminated commodities from entering the human food chain, but low-level contamination is unavoidable and remains a major food safety concern.

Regarding their toxicity, trichothecenes interfere with protein synthesis, inducing cellular stress pathways, aberrant proinflammatory responses, disrupt intestinal function and interfere with growth hormone action, leading to feed refusal, vomiting and altered immune function in experimental and farm animals, with type A trichothecenes being more potent toxins than type B trichothecenes [12]. ZEN has proven hepatotoxic, immunotoxic and carcinogenic properties in several animal species and is a potent oestrogenic compound that can cause fertility issues in farm animals [6]. OTA is a potent renal carcinogen in several animal species and is also linked to kidney damage, cardiac and hepatic abnormalities, lesions of the gastrointestinal tract and lymphoid tissue, while AFB_1_ is a well-defined hepatocarcinogen in humans and animals and is linked to impaired growth and immunomodulation in children [13]. To help assess the potential human health risk of frequent, low-level exposure to toxic compounds, health-based guidance values (HBGV) are established for many food contaminants, including mycotoxins. The tolerable daily intake (TDI) of a contaminant is identified as the amount that can be consumed over a lifetime without presenting an appreciable risk to health. TDI values are derived from toxicity studies in animals and are used to characterise a potential risk posed by a given exposure scenario. TDI values have been set by EFSA for DON and its modified forms (1 µg/kgBW/d [14]), NIV and its modified forms (1.2 µg/kgBW/d [15]), ZEN and its modified forms (0.25 µg/kgBW/d [16]), T-2/HT-2 and their modified forms (0.02 µg/kgBW/d [17]) and a Tolerable Weekly Intake (TWI) for OTA (120 ng/kgBW/d). However, a recent re-evaluation of OTA [18] has concluded that it was no longer appropriate to use the TWI and proposed a Margin of Exposure (MOE) approach for OTA instead. Reference points were set for OTA as the Benchmark Dose Lower Confidence Limit (BMDL_10_) of 4.73 µg/kgBW/d (for non-neoplastic kidney lesions) and 14.5 µg/kgBW/d (for neoplastic kidney tumours) and MOE of ≥200 and ≥10,000 considered low health concern for non-neoplastic and neoplastic effects, respectively [18].

To characterise the potential health risks of mycotoxins, a detailed understanding of the exposure that occurs in a population is critical. Quantifying exposure through a range of different food groups and diet patterns is challenging as mycotoxin contamination is heterogeneous within each commodity, and diet patterns vary greatly from one day to the next [7]. Indirect assessment of exposure relies on the excretion of mycotoxin metabolites in urine as biomarkers of exposure [19]. For this approach to accurately quantify exposure, a detailed understanding of the toxicokinetics of each mycotoxin is needed. Much of this information is based on animal studies generating urinary clearance rates of mycotoxins into urine, although some evidence from human studies is also available [20]. Analysis of human urine has generated a detailed understanding of mycotoxin metabolism in humans and identified free mycotoxins and glucuronidated hepatic metabolites as major routes of mycotoxin elimination. Hence, these compounds are frequently used as exposure biomarkers in adults and children [19]. Infants and young children are deemed at particular risk of high exposure due to their immature detoxification system and potentially high intake of contaminated foods in relation to body weight [21]. A recent detailed study assessing the exposure to a wide range of chemical food contaminants in Austrian children found that only mycotoxin exposure frequently exceeded HBGVs [22,23]. Hence, it is crucial to better understand the extent of mycotoxin exposure and potential risk in this vulnerable group.

This study therefore aims to assess the exposure and co-exposure of UK children to multiple potent mycotoxins, characterise the risk posed by dietary mycotoxin exposure and identify food groups that contribute to risk.

## 2. Results

### 2.1. Occurrence of Mycotoxin Biomarkers in Urine and Correlation with Food Consumption

#### 2.1.1. Prevalence and Range of Mycotoxins in Urine Samples

DON was the most prevalent mycotoxin found in paediatric urine samples (95.61% of samples >LOD), followed by OTA, ZEN and HT-2 toxin (Table 1). DON was also detected at the highest levels (12.10 μg/d) while excretion of OTA and ZEN was low. T-2 and HT-2 toxins were detected at 0.83 and 1.87 μg/d, respectively, while low-prevalence mycotoxins such as DOM-1, AFB_1_ and AFM_1_ were detected in 1–2 samples at high concentrations (2.68–4.96 μg/d).

#### 2.1.2. Co-Occurrence of Multiple Mycotoxins in the Same Urine Sample

**Figure 1:**
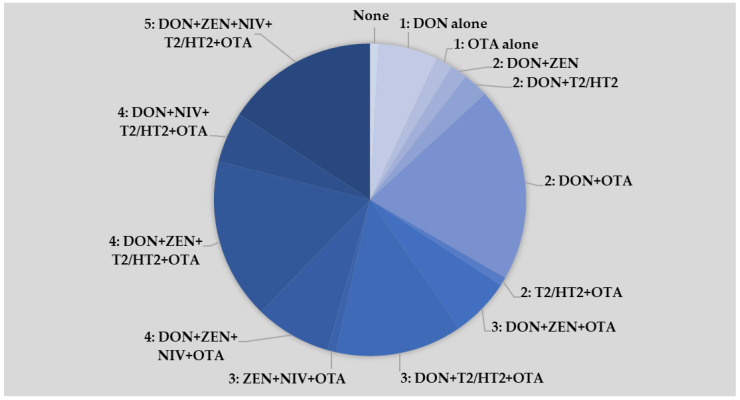
Co-occurrence of multiple mycotoxins in the same urine sample. Analysis is based on creatinine-adjusted urine concentrations and lower-bound (LB) excretion estimates (values <LOD and <LOQ assumed as 0). LOD = Limit of Detection, LOQ = Limit of Quantification.

Co-occurrence of multiple mycotoxins was frequently observed in the present study, with 25% of all samples testing positive for two mycotoxins in different combinations (DON + OTA most frequent, Figure 1). A total of 20% of samples contained three mycotoxins, 30% contained four mycotoxins and 16% contained all five major mycotoxins, with DON being present in most samples. Only 1% of samples did not have detectable levels of any mycotoxin tested.

#### 2.1.3. Association of Mycotoxin Excretion with Food Intake

Urinary mycotoxin excretion (μg/d, averaged over four days of urine sample collection for each child) was assessed for any association with food intake of food groups known to be at high risk of mycotoxin contamination. Food groups included total cereal foods (Table 2), total wholegrain foods (wholemeal bread + wholegrain and high-fibre breakfast cereals), whole-grain and high-fibre breakfast cereals, breads, oats (for T-2/HT-2) and cheese, pork and fruits for OTA (see Section 5.3 for details). Four models of food intake were evaluated (Model I: food intake on the same day as urine sampling; Model II: food intake the day before urine sampling; Model III: average food intake on the same day and day before urine sampling; Model IV: 3-day average food intake) as proposed by Turner [25]. Total cereal food intake on the same day as urine sampling (Model I) was positively associated with urinary excretion of DON, NIV and T-2/HT2 (*R*^2^ 0.188, 0.124 and 0.217, respectively, Table 2), while a weaker association was found for the average intake on the same day and previous day (Model III). This is in line with Turner [25], who reports the strongest association between total cereal food intake and DON in UK adults using Models I and III (*R*^2^ 0.22, 0.27, respectively). Urinary OTA excretion in the present study was significantly associated with total cereal food intake (Models II, III and IV, Table 2). On the contrary, no significant associations between total cereal food intake and urinary DON or serum OTA biomarkers were found in Swedish adolescents [23].

Urinary DON excretion was positively associated with total whole-grain intake (wholemeal bread + whole-grain and high-fibre breakfast cereals) on the same day (Model I *R*^2^ 0.115, Supplementary Table S1). These findings are again in line with results in UK adults [25] and Swedish adolescents [23].

T-2/HT-2 excretion was positively associated with total whole-grain intake (wholemeal bread + whole-grain and high-fibre breakfast cereals) on the same day (Model I *R*^2^ 0.286) and was strongly associated with oat intake in all models (Model I *R*^2^ 0.355, Model II *R*^2^ 0.528, Model III *R*^2^ 0.470, Model IV *R*^2^ 0.465, Table 1). This association has, to our knowledge, not been reported in biomonitoring studies before. NIV and OTA excretion were also significantly associated with oat intake in some of the models (Table 1). No significant associations were found for bread intake with any mycotoxin or for cheese, pork and fruit intake with OTA, and ZEN excretion was not associated with the intake of any food group (Supplementary Table S1).

### 2.2. Dietary Mycotoxin Exposure Assessment and Risk Characterisation

#### 2.2.1. Probable Daily Intake Estimates

Probable daily intakes (PDIs) were calculated for all mycotoxins based on creatinine-adjusted urine concentrations and different substitution methods for left-censored data (lower bound, LB; middle bound, MB; upper bound, UB [26]). Exposure to DON was calculated as DON equivalents (sum of DON+DOM-1) and exposure to ZEN as ZEN equivalents (sum of ZEN+α-ZEL+β-ZEL). The average PDI for DON equivalents was 0.89 µg/kgBW/d for all substitution scenarios due to the high prevalence of DON in urine, while the average PDI for NIV ranged from 0.29 to 0.66 µg/kgBW/d for LB and UB scenarios, respectively (Table 3). Values for two scenarios of urinary excretion rate were calculated for ZEN equivalents (based on urinary clearance rates (CRs) of 9.4 and 36.8%) and OTA (based on urinary clearance rates of 2.5 and 5.0%) to allow comparison with the literature.

#### 2.2.2. Risk Characterisation of DON, NIV, ZEN and T-2/HT2 Exposure Using Hazard Quotients (HQs)

**Figure 2:**
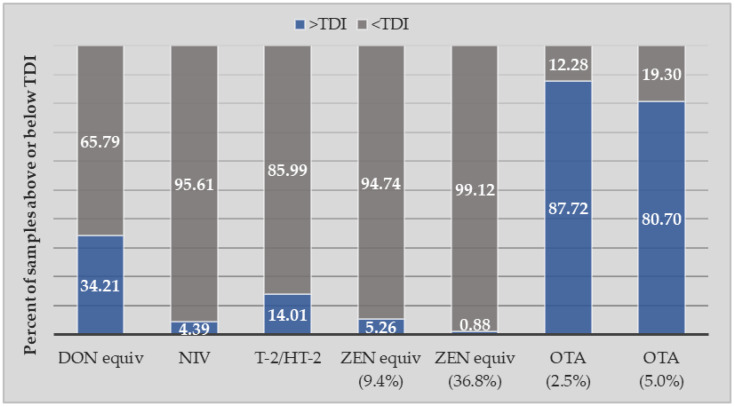
Prevalence (% of all samples, *n* = 114) above or below the tolerable daily intake (TDI) for each mycotoxin based on lower-bound (LB) exposure estimates.

Comparing PDI with tolerable daily intakes (TDIs) for each mycotoxin, HQs were calculated for LB, MB and UB scenarios (Table 3). The average HQ for DON was 0.89, and TDI exceedances were frequently observed (34.21% of all samples, Figure 2). The risk of NIV exposure was found to be low (average HQ 0.24–0.55 under LB and UB scenarios), and only 4.39% of samples exceeded the TDI. T-2/HT-2 toxins were frequently detected (60% of samples), resulting in an average PDI of 0.02 µg/kgBW/d. T-2/HT-2 exposure posed a moderate risk to children with an average HQ of 0.74–0.80 (LB-UB) and 14.01% TDI exceedances (Figure 2). For ZEN equivalents, both exposure scenarios (based on different urinary clearance rates) showed a low health risk in children with average HQ (LB) of 0.2 or 0.05 and 5.26 or 0.88% of TDI exceedances for the 9.4% and 36.8% CR scenarios, respectively.

#### 2.2.3. Risk Characterisation of OTA Exposure Using Hazard Quotient and MOE Approaches

**Figure 3:**
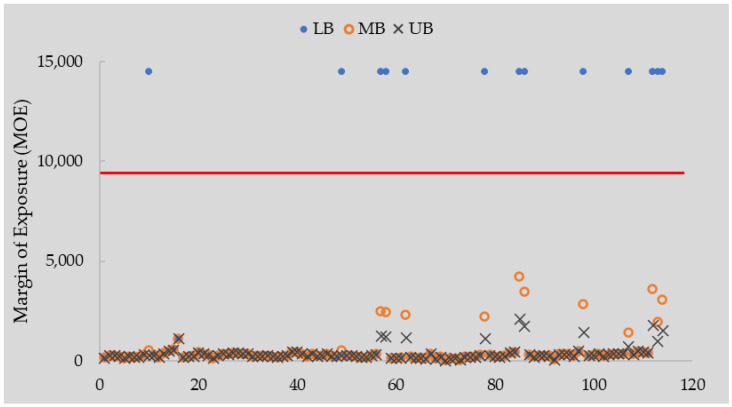
Margin of Exposure (neoplastic effects) for ochratoxin A based on 114 urine samples using LB, MB and UB exposure estimates and urinary clearance rate of 2.5% for OTA. To visualise MOE for all samples, OTA exposure was set at 0.001 µg/kgBW/d for all samples with OTA < LOD in LB scenario, resulting in MOE of 14,500 [24].

Until recently, the risk of OTA exposure was characterised using the TWI of 120 ng/kgBW, while EFSA currently recommends that no such HBGV can be established for OTA [18] and MOE approaches should be adopted instead using non-neoplastic and neoplastic reference points. We have adopted both approaches in our analysis and found a high mean HQ for OTA using LB exposure estimates (4.19 and 2.10 based on 2.5% and 5% CR scenarios, Table 3) and a high risk of TWI exceedances (87.72%, Figure 2). Similarly, when comparing exposure to the non-neoplastic reference point, we found narrow Margins of Exposure (mean 65.83) in 87.82% of samples under the LB scenario, indicating a potential health concern (Table 4). For neoplastic lesions, OTA exposure resulted in narrow MOE (mean 201.82) in 88.60% of cases in the LB exposure scenario, which increased to 100% of cases in MB and UB scenarios (Table 4, Figure 3). These findings across different risk assessment approaches clearly demonstrate the potential risk posed by OTA exposure in children.

When examining co-exposure to several mycotoxins at high levels, two cases (1.75% of urine samples) were found where all five major mycotoxins exceeded the safe level of intake (either HQ > 1 or MOE < 200). These samples were from two children in separate families. In the majority of cases, unsafe high-level exposure was limited to one mycotoxin (53.51% of cases, mainly OTA alone) or two mycotoxins (26.32% of cases, mainly DON+OTA). Only 8.77% of cases did not exceed the safe level of intake of any mycotoxin.

## 3. Discussion

The current study aimed to quantify mycotoxin exposure in children and characterise the risk arising from such exposure. DON exposure was frequently detected in UK children (>95% of samples), with the average probable daily intake (PDI) estimated to be below the tolerable daily intake (TDI). However, 34% of PDI estimates from the current study exceeded the TDI, which is similar to a previous report on UK children (33–50% > TDI, [33]). One large paediatric study in Belgium reports even higher DON exposure and more frequent TDI exceedances (69% > TDI, [34]), while other European studies report low risk posed by DON exposure (22% > TDI in Austrian children [23], 10% > TDI in Norwegian children [35], 12.5% > TDI in Spanish children [36] and 1.6% in Swedish adolescents [24]). The frequent TDI exceedances in U.K. children warrant attention from risk managers, especially during years of high risk for *Fusarium* infection when exposure risk is increased [4].

Nivalenol is rarely reported in mycotoxin biomonitoring studies as this compound is either not assessed [24,34] or reported below the Limit of Quantification [23,36]. The present study, which utilised immunoaffinity column enrichment to lower the Limit of Quantification, found NIV to be detectable in 39% of urine samples and quantifiable in 26%. Based on our findings, TDI exceedances are rare in UK children (4% of samples), whereas [28] reports the mean PDI in European adults to exceed the TDI by 35-fold. Given the small number of studies and high uncertainty related to the low urinary clearance rate for NIV (1% in rats), this potential risk urgently requires more attention.

The current study reports frequent exposure to T-2/HT-2 (60% of samples), with 14.0% of PDI estimates exceeding the TDI. In other studies, T-2/HT-2 exposure was either assessed but not detected [23,34] or reported in 0.1% of samples below LOQ in adolescents in Sweden [24]. On the contrary, HT-2 exposure was reported in 6% of Spanish paediatric urine samples at high concentrations (12.6 ng/mg creatinine, which is close to our maximum level [36]), and a study in Italian children reported a high prevalence (20 and 32%) at moderate levels (1.26 and 1.19 ng/mg creatinine) for T-2 and HT-2 [37]. Based on food analysis rather than biomarker analysis, a French total diet study found that children are exposed to an average of 14.5 ng T-2/HT-2/kgBW/d, indicating a significant risk of exceeding safe levels of exposure (TDI 20 ng/kgBW/d) [7]. These findings, together with the high average PDI estimated for European adults at approximately 10 times the TDI [28], highlight a potential health risk that urgently requires further studies capable of detecting low levels of T-2/HT-2 in human biofluids.

ZEN exposure is frequently reported, but the risk associated with dietary ZEN appears negligible in the current study as well as in published studies in children [23,34,36] and adults, especially in Northern Europe [28].

The potential risk posed by OTA exposure was assessed by two approaches in this study, comparing PDI estimates either to TDI or calculating Margins of Exposure (MOE) for non-neoplastic and neoplastic effects. Compared to TDI, we found an average HQ to indicate a significant risk due to OTA exposure. When adopting the MOE approach, we found a narrow MOE, indicating a health risk for neoplastic effects in all children and for non-neoplastic effects in most children. These findings are in agreement with findings in Austrian children (mean MOE 184 for non-neoplastic and 565 for neoplastic effects, [23]) and with the large-scale European study in adults (average MOE 14–48 for non-neoplastic effects, [28]). Our findings across different risk assessment approaches clearly demonstrate the risk posed by OTA exposure in children.

Mycotoxin exposure in children poses an even greater risk in other parts of the world where contamination of breast milk, milk and cereal foods for infants has been found to be contaminated with a range of potent mycotoxins and exposure confirmed in biomonitoring studies using blood and urine [38].

Co-exposure to multiple mycotoxins has been frequently found in the current study, as well as in some recent biomonitoring studies [23,39]. Given the frequent co-contamination of food items with multiple mycotoxins and the complex nature of the human diet, it is not surprising that co-exposure is common [40]. However, the assessment of the combined toxic effects of chemical mixtures and characterisation of risk posed by exposure to multiple contaminants is currently poorly understood.

In urinary biomonitoring studies, a range of scenarios can be employed to estimate dietary exposure and assess the associated risk in a study population. Firstly, left-centred urinary mycotoxin data can be processed in different ways, calculating lower-bound, middle-bound and upper-bound scenarios for mycotoxin levels below the Limit of Detection and quantification of a given method [26]. This approach affects mycotoxins with lower prevalence to a greater extent, such as NIV in the present study. Secondly, mycotoxin levels are corrected for urinary clearance rate to extrapolate dietary intake. The urinary clearance rate for DON is well established from papers in humans [27,29], while the NIV clearance rate of 1% is based on one rodent study [28]. For ZEN (9.4 and 36.8%) and OTA (2.5, 5 or 50%), different clearance rates have been used, and great care is needed when comparing published results. Employing a high clearance rate will lead to a drastic underestimation of risk and highlights the urgent need to harmonise approaches to risk assessment to allow meaningful interpretation of findings and risk characterisation across different populations.

This study reports, for the first time, that urinary DON excretion is significantly associated with total cereal intake, total wholegrain food intake and wholegrain breakfast cereal intake in UK children, confirming previous findings in UK adults [25] and Swedish adolescents [24]. We also report, for the first time, that total cereal intake is positively associated with urinary NIV, T-2/HT-2 and OTA excretion and that total whole-grain food intake and oat intake are positively associated with urinary T-2/HT-2 excretion in children.

## 4. Conclusions

This urinary mycotoxin biomonitoring study in children demonstrates the frequent exposure of children to multiple important dietary mycotoxins. Trichothecenes, zearalenone and ochratoxin A were frequently detected, and co-exposure to multiple mycotoxins was found in 91% of urine samples. In conclusion, our work demonstrates for the first time that UK children are frequently exposed to multiple mycotoxins through their diet and that cereal foods contribute strongly to exposure. The probable daily intake estimates of some mycotoxins (DON and OTA) are high enough to pose a health concern if exposure is continuous.

## 5. Materials and Methods

### 5.1. Study Design

A total of 29 children (16 girls and 13 boys, aged 2–6 years) from 20 families were recruited into this study, and their anthropometry data are summarised in Table 5. All children followed their normal habitual diet. This study was approved by the Rowett Institute’s Ethics Review Panel following favourable consideration by the Grampian Research Ethics Committee (Reference 01/0306).

### 5.2. Urine Mycotoxin Analysis and Calculation of Mycotoxin Excretion

Spot urine samples were collected from 28 children on four consecutive days during the period that food diaries were kept. One child only provided two urine samples. Samples were collected in 2004 and stored in the Rowett Institute biorepository at −20 °C until analysis. Urine samples (*n* = 114) were analysed for 11 mycotoxins: deoxynivalenol (DON), de-epoxy-deoxynivalenol (DOM-1), nivalenol (NIV), HT-2 toxin (HT-2), T-2 toxin (T-2), ochratoxin A (OTA), zearalenone (ZEN), α-zearalenol (α-ZEL), β-zearalenol (β-ZEL), aflatoxin B_1_ (AFB_1_) and aflatoxin M_1_ (AFM_1_). The extraction method and LC-MS/MS method performance parameters are described in detail in [41]. In brief, urine samples were spiked with a mix of stable-isotope labelled internal standards (^13^C_15_ DON, ^13^C_22_ HT-2; ^13^C_18_ ZEN, ^13^C_20_ OTA, ^13^C_17_ AFB_1_), digested overnight with β-glucuronidase (Sigma-Aldrich, Ltd., Pool, UK), enriched through immunoaffinity columns (Myco-6in1, Vicam V100000176, Biocheck, St Asaph, UK) and eluted into methanol. The liquid chromatography separation of mycotoxins was performed on a Shimadzu Nexera X2 LC system using an Agilent Poroshell column. The LC eluant was directed into a Shimadzu 8060 triple-quadrupole MS. Mycotoxins were quantified using the multiple reaction monitoring technique. Limit of Detection (LOD) and Limit of Quantification (LOQ) for all mycotoxins are quoted in Table 1. Urinary creatinine levels were measured using an alkaline picrate solution on an automated clinical analyser (KONELAB 30, Labmedics, Stockport, U.K.).

The daily urinary creatinine clearance (mg/day) was estimated as a function of body weight, age and gender:

Creatinine excretion female (mg/d) = (22 − age/9) × bodyweight

Creatinine excretion male (mg/d) = (28 − age/6) × bodyweight

Daily urinary mycotoxin excretion (μg/d) was calculated as follows:

Urinary mycotoxin (ng/mg creatinine) × total 24 h creatinine excretion (mg/d)

Daily urinary mycotoxin excretion (ug/d) was calculated using lower-bound (LB), middle-bound (MB) and upper-bound (UB) scenarios [24]. For LB scenarios, all values <LOD and <LOQ are assumed to be 0. For MB scenarios, all values <LOD are assumed as ½ LOD, and all values <LOQ are assumed as ½ LOQ. For UB scenarios, all values <LOD are assumed as LOD, and all values <LOQ are assumed as LOQ.

### 5.3. Analysis of Food Consumption Data and Association with Urinary Mycotoxins

Daily food intake was recorded using food diaries for 7-14 consecutive days, during which the four spot urine samples were collected. Food diaries were analysed using WinDiets Nutritional Analysis Software Suite version 1.0; the food list comprises 4082 items of foods and drinks, which were grouped into food groups and subgroups according to the UK National Diet and Nutrition Survey [42]. Total cereal foods included pasta, pizza, bread, breakfast cereals, biscuits, buns, cakes, pastries, pies, cereal-based puddings, sponge puddings and other cereals. Total wholegrain foods include wholemeal bread + wholegrain and high-fibre breakfast cereals. Wholegrain breakfast cereals include wholegrain and high-fibre breakfast cereals, porridge and muesli. Breads include white bread, brown and granary bread, wheatgerm bread, wholemeal bread and other bread. Oats include oatcakes, oat-based cereal bars, porridge, muesli and oatmeal. Cheese includes cottage cheese, cheddar and other cheese. Pork includes bacon and ham, pork and pork dishes and sausages. Fruits include grapes, grape juice, sultanas, raisins, dried apricots, dried pineapples and dried mixed fruit. Total daily food intake (g/d) for high-risk food groups was calculated for each child on the same day as urine sampling (Model I), the day before urine sampling (Model II), the average of the same day and the day before urine sampling (Model III) and the average intake across three days during and before urine sampling (Model IV) as proposed by [25].

The average food intake calculated by using the four models (described above) and the urinary mycotoxin excretion (DON, ZEN, NIV, T-2/HT-2 and OTA) calculated per urine sample were then averaged to obtain a single value per child. Repeated measure analysis looking at food intake across four urine days found no significant association. The effect of food intake on urine mycotoxin levels for the various models was analysed using a univariate linear model. The presence of any association is indicated by a *p*-value < 0.05. All analyses were conducted in R version 4.3.1.

### 5.4. Dietary Exposure Assessment and Risk Characterisation

The dietary mycotoxin exposure (probable daily intake, PDI) is calculated as follows:

Daily urinary mycotoxin excretion (μg/d)/urinary clearance rate % (Table 3)

Risk characterisation was performed by comparing PDI values with tolerable daily intake (TDI) for DON NIV, T-2/HT-2, ZEN and OTA. Additionally, a Margin of Exposure approach was used to compare the OTA PDI with the reference value (Benchmark Dose Lower 10) for non-neoplastic (4.73 μg/kgBW/d) and neoplastic lesions (14.5 μg/kgBW/d) [18].

MOE was calculated as follows:

Reference value (μg/kgBW/d)/mycotoxin intake (μg/kgBW/d)

MOE values > 200 are considered safe for compounds with non-neoplastic effects, and values > 10,000 are considered safe for compounds with neoplastic effects.

## Figures and Tables

**Table 1:** Urinary excretion of multiple mycotoxins in 29 children providing 114 urine samples.

Mycotoxin (LOD/LOQ) ^1^	Samples >LOD	Samples >LOQ	Mean (Range) of Positive Samples ^2^	Mean (Range) of Positive Samples ^2^	Mean (Range) of Positive Samples ^2^
ng/mL Urine	N (%)	N (%)	ng/mL Urine	ng/mg Creatinine	μg/d
**DON** (0.156/0.328)	109 (95.61)	109 (95.61)	13.24 (0.65/56.08)	28.47 (1.44/177.27)	12.10 (0.67/55.17)
**DOM-1** (0.063/0.158)	2 (1.75)	2 (1.75)	0.35 (0.21/0.48)	0.80 (0.24/1.36)	3.28 (0.75/5.81)
**NIV** (0.066/0.125)	45 (39.47)	30 (26.32)	0.17 (0.06/0.58)	0.36 (0.07/3.52)	0.14 (0.02/1.10)
**HT-2** (0.013/0.033)	64 (56.14)	60 (52.63)	0.19 (0.02/1.77)	0.65 (0.02/15.48)	1.87 (0.06/49.73)
**T-2** (0.006/0.013)	11 (9.65)	11 (9.65)	0.08 (0.02/0.55)	0.24 (0.03/1.55)	0.83 (0.07/6.63)
**OTA** (0.003/0.006)	103 (90.35)	101 (88.60)	0.04 (0.00/0.52)	0.09 (0.02/1.49)	0.03 (0.01/0.35)
**ZEN** (0.016/0.033)	89 (78.07)	55 (48.25)	0.06 (0.02/0.46)	0.13 (0.02/1.35)	0.05 (0.01/0.42)
**α-ZEL** (0.033/0.063)	13 (11.40)	13 (11.40)	0.21 (0.11/0.56)	0.55 (0.14/1.60)	1.60 (0.34/6.85)
**β-ZEL** (0.033/0.063)	13 (11.40)	13 (11.40)	0.19 (0.10/0.63)	0.39 (0.10/1.81)	1.23 (0.13/7.73)
**AFB_1_** (0.003/0.006)	1 (0.88)	1 (0.88)	0.41	1.16	4.96
**AFM_1_** (0.003/0.006)	2 (1.75)	1 (0.88)	0.22 (0.00/0.44)	0.63 (0.00/1.25)	2.68 (0.01/5.34)

^1^ LOD = Limit of Detection, LOQ = Limit of Quantification. ^2^ To calculate averages of positive samples, values <LOD were removed, and values >LOD<LOQ were replaced with ½ LOQ [24]. DON (deoxynivalenol), DOM-1 (de-epoxy deoxynivalenol), NIV (nivalenol), HT-2 (HT-2 toxin), T-2 (T-2 toxin), OTA (ochratoxin A), ZEN (zearalenone), α-ZEL (α-zearalenol), β-ZEL(β-zearalenol), AFB_1_ (aflatoxin B_1_), AFM_1_ (aflatoxin M_1_).

**Table 2:** Association of mycotoxin excretion with total cereal food and oat intake.

	DON Equiv		NIV		T-2/HT-2		OTA	
Food Intake (Model ^1^)	Coefficient (SE)	*R*^2^ (*p*-Value)	Coefficient (SE)	*R*^2^ (*p*-Value)	Coefficient (SE)	*R*^2^ (*p*-Value)	Coefficient (SE)	*R*^2^ (*p*-Value)
**Cereals** **(I)**	0.051 (0.019)	0.188 * (0.011)	<0.001 (<0.001)	0.124 * (0.034)	0.002 (0.001)	0.217 * (0.006)	<0.001 (<0.001)	0.095 (0.058)
**Cereals** **(II)**	0.050 (0.020)	0.162 * (0.017)	<0.001 (<0.001)	0.059 (0.108)	0.002 (0.001)	0.094 (0.059)	<0.001 (<0.001)	0.113 * (0.042)
**Cereals** **(III)**	0.052 (0.020)	0.176 * (0.014)	<0.001 (<0.001)	0.092 (0.061)	0.002 (0.001)	0.164 * (0.017)	<0.001 (<0.001)	0.107 * (0.047)
**Cereals** **(IV)**	0.049 (0.021)	0.142 * (0.025)	<0.001 (<0.001)	0.051 (0.124)	0.002 (0.001)	0.122 * (0.035)	<0.001 (<0.001)	0.112 * (0.042)
**Oats** **(I)**	0.041 (0.061)	−0.020 (0.505)	0.001 (0.001)	0.063 (0.101)	0.008 (0.002)	0.355 * (<0.001)	0.001 (<0.001)	0.262 * (0.003)
**Oats** **(II)**	0.046 (0.067)	−0.019 (0.492)	0.002 (0.001)	0.228 * (0.005)	0.011 (0.002)	0.528 * (<0.001)	<0.001 (<0.001)	0.056 (0.114)
**Oats** **(III)**	0.047 (0.066)	−0.018 (0.487)	0.001 (0.001)	0.148 * (0.022)	0.010 (0.002)	0.470 * (<0.001)	0.001 (<0.001)	0166 * (0.016)
**Oats** **(IV)**	0.040 (0.069)	−0.024 (0.565)	0.001 (0.001)	0.160 * (0.018)	0.011 (0.002)	0.465 * (<0.001)	<0.001 (<0.001)	0.131 * (0.030)

* significant results (*p*-Value < 0,05); Middle-bound (MB) estimates for urine mycotoxin excretion were correlated with food intake. ^1^ Model I (food intake on same day as urine sampling), Model II (food intake the day before urine sampling), Model III (average food intake on same day and day before urine sampling), Model IV (average food intake across 3 days) as in [25]. Analysis is based on 29 children. Details on intake of each food groups and complete results for all food groups, Models I–IV and lower-bound (LB), middle-bound (MB) and upper-bound (UB) mycotoxin estimates are presented in Supplementary Table S1.

**Table 3:** Probable daily intake (PDI) of mycotoxins estimated from creatinine-adjusted urine concentrations and risk assessment using tolerable daily intakes (TDIs).

	PDI for Mycotoxins (µg/kgBW/d)			Risk Characterisation (Hazard Quotient PDI/TDI ^1^)		
Mycotoxin	LB ^2^	MB ^3^	UB ^4^	LB ^2^	MB ^3^	UB ^4^
(Clearance Rate ^5^)	Mean (Range)	Mean (Range)	Mean (Range)	Mean (Range)	Mean (Range)	Mean (Range)
**DON equiv** (72.3% [27])	0.89 (0.00/5.30)	0.89 (0.00/5.30)	0.89 (0.01/5.30)	0.89 (0.00/5.30)	0.89 (0.00/5.30)	0.89 (0.01/5.30)
**NIV** (1.0% [15])	0.29 (0.00/7.61)	0.47 (0.06/7.61)	0.66 (0.12/7.61)	0.24 (0.00/6.34)	0.39 (0.05/6.34)	0.55 (0.10/6.34)
**T-2/HT-2** (60.0% [28])	0.01 (0.00/0.56)	0.02 (0.00/0.56)	0.02 (0.00/0.56)	0.74 (0.00/27.88)	0.77 (0.02/27.92)	0.80 (0.03/27.97)
**ZEN equiv** (9.4% [29])	0.05 (0.00/1.08)	0.06 (0.00/1.08)	0.06 (0.00/1.08)	0.20 (0.00/4.33)	0.22 (0.01/4.33)	0.24 (0.02/4.33)
**ZEN equiv** (36.8% [30])	0.01 (0.00/0.28)	0.01 (0.00/0.28)	0.02 (0.00/0.28)	0.05 (0.00/1.11)	0.06 (0.00/1.11)	0.06 (0.00/1.11)
**OTA** (2.5% [31,32])	0.07 (0.00/1.29)	0.07 (0.00/1.29)	0.07 (0.01/1.29)	4.19 (0.00/75.17)	4.25 (0.20/75.17)	4.31 (0.40/75.17)
**OTA** (5.0% [31])	0.04 (0.00/0.64)	0.04 (0.00/0.64)	0.04 (0.00/0.64)	2.10 (0.0/37.59)	2.13 (0.10/37.59)	2.16 (0.20/37.59)

^1^ TDI = 1.0 (DON), 1.2 (NIV), 0.02 (T-2+HT-2), 0.25 (ZEN) and 0.017 (OTA, derived from TWI of 0.12) µg/kgBW/d. ^2^ LB = lower bound assuming all values <LOD and <LOQ as 0. ^3^ MB = middle bound, assuming all values <LOD as ½ LOD and <LOQ as 1/2LOQ. ^4^ UB = upper bound assuming all values <LOD as LOD and <LOQ as LOQ. ^5^ Different urinary clearance rates (CRs) were derived from the literature.

**Table 4:** Risk characterisation of OTA based on Margin of Exposure approaches using Benchmark Dose Lower _10_ for non-neoplastic and neoplastic lesions.

Mycotoxin		Average Margin of Exposure (Calculated from Average PDI)			% of Samples <MOE		
(Clearance Rate)	Margin of Exposure	LB	MB	UB	LB	MB	UB
**OTA** (2.5%)	200 (non-neoplastic) ^1^	65.83	64.89	63.97	87.82	89.47	89.47
**OTA** (2.5%)	10,000 (neoplastic) ^2^	201.82	198.92	196.10	88.60	100.00	100.00

^1^ based on Benchmark Dose Lower (BMDL_10_) of 4.73 µg/kgBW/d (non-neoplastic). ^2^ based on Benchmark Dose Lower (BMDL_10_) of 14.5 µg/kgBW/d (neoplastic). LB = lower bound assuming all values <LOD and <LOQ as 0. MB = middle bound, assuming all values <LOD as ½ LOD and <LOQ as 1/2LOQ. UB = upper bound assuming all values <LOD as LOD and <LOQ as LOQ.

**Table 5:** Anthropometry information of children.

Gender	Age (yrs)	Body Weight (kgs)	Urinary Creatinine (mg/d)
Boys, *n* = 13	4.52 ± 1.34	18.46 ± 2.57	437.18 ± 83.96
Girls, *n* = 16	4.24 ± 1.27	17.65 ± 6.12	413.46 ± 142.49
Total, *n* = 29	4.37 ± 1.29	18.01 ± 4.80	424.09 ± 118.50

Values expressed as mean ± standard deviation.

## Data Availability

Data will be made available on reasonable request to the corresponding author.

## Supplementary Materials

The following supporting information can be downloaded at https://www.mdpi.com/article/10.3390/toxins16060251/s1, Table S1: Associations between urinary mycotoxin excretion and food intake using Models I-IV and lower-bound (LB), middle-bound (MB) and upper-bound (UB) exposure estimates.

## Abbreviations

Deoxynivalenol (DON), nivalenol (NIV, T-2 toxins (T-2), HT-2 toxin (HT-2)), zearalenone (ZEN), zearalenol (ZEL), ochratoxin A (OTA), aflatoxin B_1_ (AFB_1_), European Food Safety Authority (EFSA), probable daily intake (PDI), tolerable daily intake (TDI), Benchmark Dose Lower (BMDL), health-based guidance value (HBGV), Margin of Exposure (MOE), Limit of Detection (LOD), Limit of Quantification (LOQ), lower bound (LB), middle bound (MB), upper bound (UB), Hazard Quotient (HQ).
